# Insights Into the Microbiology of the Chaotropic Brines of Salar de Atacama, Chile

**DOI:** 10.3389/fmicb.2019.01611

**Published:** 2019-07-11

**Authors:** Carolina F. Cubillos, Adrián Paredes, Carolina Yáñez, Jenifer Palma, Esteban Severino, Drina Vejar, Mario Grágeda, Cristina Dorador

**Affiliations:** ^1^Laboratorio de Complejidad Microbiana y Ecología Funcional, Instituto Antofagasta, Universidad de Antofagasta, Antofagasta, Chile; ^2^Department of Chemical Engineering and Mineral Process, Center for Advanced Study of Lithium and Industrial Minerals, Universidad de Antofagasta, Antofagasta, Chile; ^3^Centre for Biotechnology and Bioengineering, Universidad de Chile, Santiago, Chile; ^4^Laboratorio Química Biológica, Instituto Antofagasta, Universidad de Antofagasta, Antofagasta, Chile; ^5^Departamento de Química, Facultad de Ciencias Básicas, Universidad de Antofagasta, Antofagasta, Chile; ^6^Laboratorio Microbiología, Instituto de Biología, Facultad de Ciencias, Pontificia Universidad Católica de Valparaíso, Valparaíso, Chile; ^7^Departamento de Ciencias de los Alimentos, Facultad de Ciencias de la Salud, Universidad de Antofagasta, Antofagasta, Chile; ^8^Departamento de Biotecnología, Facultad de Ciencias del Mar y Recursos Biológicos, Universidad de Antofagasta, Antofagasta, Chile

**Keywords:** lithium-tolerance, bacteria, halophiles, *Bacillus*, Lithium Triangle Zone

## Abstract

Microbial life inhabiting hypersaline environments belong to a limited group of extremophile or extremotolerant taxa. Natural or artificial hypersaline environments are not limited to high concentrations of NaCl, and under such conditions, specific adaptation mechanisms are necessary to permit microbial survival and growth. Argentina, Bolivia, and Chile include three large salars (salt flats) which globally, represent the largest lithium reserves, and are commonly referred to as the Lithium Triangle Zone. To date, a large amount of information has been generated regarding chemical, geological, meteorological and economical perspectives of these salars. However, there is a remarkable lack of information regarding the biology of these unique environments. Here, we report the presence of two bacterial strains (isolates LIBR002 and LIBR003) from one of the most hypersaline lithium-dominated man-made environments (total salinity 556 g/L; 11.7 M LiCl) reported to date. Both isolates were classified to the *Bacillus* genera, but displayed differences in 16S rRNA gene and fatty acid profiles. Our results also revealed that the isolates are lithium-tolerant and that they are phylogenetically differentiated from those *Bacillus* associated with high NaCl concentration environments, and form a new clade from the Lithium Triangle Zone. To determine osmoadaptation strategies in these microorganisms, both isolates were characterized using morphological, metabolic and physiological attributes. We suggest that our characterization of bacterial isolates from a highly lithium-enriched environment has revealed that even at such extreme salinities with high concentrations of chaotropic solutes, scope for microbial life exists. These conditions have previously been considered to limit the development of life, and our work extends the window of life beyond high concentrations of MgCl_2_, as previously reported, to LiCl. Our results can be used to further the understanding of salt tolerance, most especially for LiCl-dominated brines, and likely have value as models for the understanding of putative extra-terrestrial (e.g., Martian) life.

## Introduction

The survival and growth of living organisms in any environment is controlled by biotic and abiotic factors (Kristjánsson and Hreggvidsson, 1995). From an anthropocentric point of view, extreme environments can be defined as habitats where one or more abiotic conditions extend beyond the typical physiological tolerances of most higher organisms (Wiegel and Canganella, 2011), e.g., low or high temperatures, extremes of pH, high concentrations of metals or salts (Peoples and Bartlett, 2017). Organisms that can live in such hostile habitats are typically microbes, and are commonly known as extremophile or extremotolerant taxa (Rodriguez-Valera et al., 1985; Orellana et al., 2018). The most commonly used classification of extremophile is related to NaCl concentrations, where taxa can be classified as either non-halophilic, halophile and extremely halophile (Tiquia et al., 2007; Lee et al., 2018). Such microorganisms are commonly found in evaporation ponds, sea waters, saline salterns, and sediments, as well as other saline habitats (Maheshwari and Saraf, 2015; Lee et al., 2018). In particular, bacteria belonging to the *Bacillales* order are found in almost every environment on Earth from the stratosphere (Sass et al., 2008), through to NaCl-hypersaline environments, such as salterns (Garabito et al., 1998), saline soils (Zahran, 1997), freshwater lakes (Bagheri et al., 2012; Amoozegar et al., 2014, 2016), and even in alkaline lakes (Weisser and Trüper, 1985). Moreover, they have been isolated from brines with different chemical composition including MgCl_2_ (Hallsworth et al., 2007; Sass et al., 2008), Na_2_CO_3_ (Jones et al., 1998), and NaCl/LiCl (Rodríguez-Contreras et al., 2013; Martínez et al., 2018). There is an increasing awareness that saline environments include both natural and artificial environments, and extend beyond those dominated by NaCl, with microbial life being reported at high salinities of other salts or even different pH as Na_2_CO_3_ (Imhoff et al., 1979; Banciu and Muntyan, 2015), CaCl_2_ (Dickson et al., 2013), MgCl_2_ (Yakimov et al., 2015), LiCl (Cubillos et al., 2018) or acid (Benison, 2008, 2019) and basic brines (Samylina et al., 2014).

Lithium is the 27th most abundant element on Earth (Aral and Vecchio-Sadus, 2008), and worldwide concentrations are ∼ 65 ppm (Habashi, 1997; Aral and Vecchio-Sadus, 2008). In nature, lithium is found in both a solid (in minerals) and liquid form, as brines in aquatic ecosystems (Wanger, 2011). An alkaline-metal belonging to Group I of the periodic table, lithium is known for its wide use, including the manufacture of glass and ceramics as well as the treatment of psychiatric disorders (Kszos et al., 2003). It is also used to determine the age of stars and the consumption of planets by these celestial bodies (Israelian et al., 2004).

The so-called Lithium Triangle Zone represents up to 85% of global reserves of soluble lithium (Martínez et al., 2018) and is formed by the Salar del Hombre Muerto (Argentina), Salar de Uyuni (Bolivia) and Salar de Atacama (Chile). Approximately 70% of all global production of lithium (approximately) is derived from Salar del Hombre Muerto (16% of Li_2_CO_3_ production) and Salar de Atacama (53% of Li_2_CO_3_ production) (Tahil, 2007; Nacif and Lacabana, 2015). Although dominated by NaCl, the brines in these salares have the highest average concentration of lithium reported from natural environments, Salar de Uyuni: 400–700 mg/L; Salar del Hombre Muerto: 773 mg/L; and Salar de Atacama: 1,500 mg/L Li (Habashi, 1997). The recent upsurge in the use of lithium as a raw material for batteries for vehicles and electronic devices has increased global demand (Wanger, 2011). The industrial process used to obtain these products begins with the pumping of natural brines (0.12% Li), which are then treated to increase lithium concentration in a sequential process of evaporation using natural solar radiation (which is extremely elevated in the region). Finally, the concentrated brines (6% Li) are used as a raw material in chemical processes to obtain lithium products (Garret, 2004). These natural Li reservoirs have been widely characterized from geological, hydrological, meteorological and economical viewpoints (Garret, 2004; Vila, 2010; An et al., 2012; Nacif and Lacabana, 2015). Microbial communities have been less studied, but have shown a recent increase in interest (Perez-Fernandez et al., 2016; Haferburg et al., 2017; Rubin et al., 2017; Cubillos et al., 2018; Martínez et al., 2018). At a microbiological level, lithium has been classified as an antimicrobial compound (Lieb, 2004), associated with the stimulation of autolysis (Sugahara et al., 1983), sporulation in *Bacillus*-species (Warburg et al., 1985; Li et al., 2014) or growth inhibition in fungi (Kurita and Funabashi, 1984) and yeasts (Morris, 2004). Nevertheless, the capacity of some bacteria to accumulate lithium by teichoic acid polymers from solution has been reported (Tsuruta, 2005; Belfiore et al., 2018).

Since the first description of dormancy in *Bacillus subtilis* (Cohn, 1872) and the recognition of *Bacillus anthracis* as the agent of anthrax (Koch, 1876), the importance of the genus *Bacillus* was recognized early in the timeline of modern science (Zeigler and Perkins, 2015). A Gram-positive, aerobic or facultative anaerobic microorganism with rod-shaped cells, *Bacillus* is among the most well-characterized microbial genera at biochemical, genomic, and proteomic levels (Alcaraz et al., 2010). The ubiquity of this microbial group has been well reported, due to its presence in a wide range of contrasting habitats including air, fresh and marine water, alkaline soils, geothermal heated soils, stone surfaces of ancient monuments, soda lakes, and cold environments (Logan and De Vos, 2015), highlighting the wide metabolic potential of *Bacillus* (Alcaraz et al., 2010). Colonial morphology in *Bacillus* varies from circular to irregular, from entire edge to undulate, with sizes ranging from 1 to 5 mm and colors from a creamy-gray to off-white (Logan and De Vos, 2015). Up until 2015, 142 *Bacillus*-species were described (Logan and De Vos, 2015), with species subclassified according to their ecophysiology as halophile, acidophiles, psychrophiles, alkalophiles, and thermophiles (Ravel and Fraser, 2005). An alternative approach to classification of *Bacillus* species reflects their use by, and their impacts on human society, including environmental (*B. subtilis*), pathogenic (*B. anthracis*), and industrial uses (*B. licheniformis*) (Alcaraz et al., 2010). Beyond their use in industry, the highly resistant endospores of *B. subtilis* have been widely studied as a model species in astrobiology (Nicholson et al., 2000). Genomic studies of *Bacillus* from different habitats (halophile, aquatic, pathogenic, deep-ocean, and soil) have revealed that 814 genes form the core genome of *Bacillus* and that 53 are related to sporulation and the competence core genome (Alcaraz et al., 2010). The high variability of this genome zone indicates that sporulation and spore resistance reflects that of the niche occupied (Alcaraz et al., 2010).

Here, we evaluated growth in the presence of lithium of two lithium-tolerant bacterial isolates obtained from a hypersaline LiCl-dominated environment (total salinity 556 g/L; 11.7 M LiCl), (Cubillos et al., 2018). Furthermore, we compared the similarities of the 16S rRNA gene of our isolates with type strains of *Bacillus* associated with NaCl and from the Lithium Triangle Zone of South America. We hypothesize that Salar de Hombre Muerto, Salar de Uyuni and Salar de Atacama support extreme, unique and unstudied microbial life capable of resisting the presence of various stressful agents including high concentrations of lithium and other salts, chaotropic activity, evaporation, and high radiation, providing a potential microbial niche. Furthermore, we suggest that this information could be important for understanding physiology at the extreme boundary of life in terrestrial or even extra-terrestrial brines such as those found on Mars (Benison and Bowen, 2006).

## Materials and Methods

### Study Site and Sampling

**FIGURE 1:**
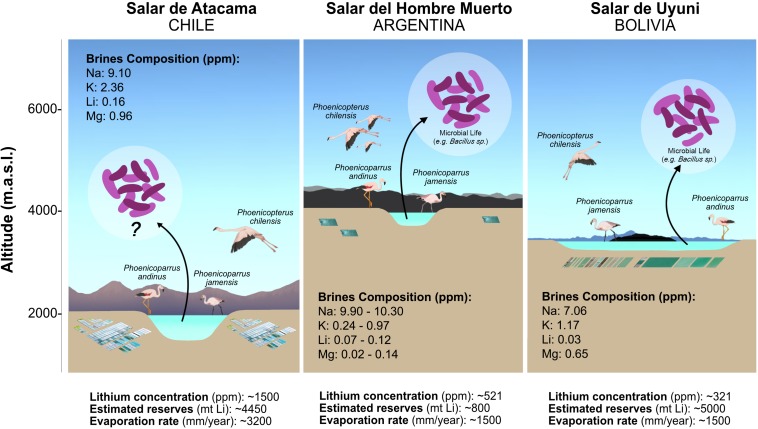
Salares belonging to the Lithium Triangle Zone, their characteristics and key studies (Hurlbert and Chang, 1983; Garret, 2004; An et al., 2012; Lorenzini, 2012; Nacif and Lacabana, 2015; Perez-Fernandez et al., 2016; Haferburg et al., 2017; Rubin et al., 2017; Cubillos et al., 2018; Martínez et al., 2018).

Concentrated lithium brines (5.8% Li) were obtained as a product of the last stage of the industrial evaporation process performed in the Salar de Atacama (Figure 1). In August 2014, 5 l of these brines were collected directly from one evaporation pool. This brine was transported (1 h) to the laboratory and then stored at 4°C for 2 days in darkness until further processing. The total dissolved salts were measured by drying the brines at 180°C (SM 2540 C) and absolute salinity was calculated from chemical analysis. Ions including lithium, magnesium, sodium, calcium, and potassium were measured by direct aspiration flame-air atomic absorption spectrophotometry (Model220FSVarian, Inc.). Sulfates were measured by gravimetry (SM 4500-SO_4_^–2^-D) and chloride through the argentometric method (SM 4500-Cl-B). The remaining chemical and physical characterizations are described by Cubillos et al., 2018.

### Enrichment, Isolation, and Identification

To obtain microbial cells from lithium concentrated brines, a series of eight different media were used to enrich cultures. Three commercial media were used: Marine Broth (BD Difco, Thermo Fisher Scientific), Tryptic Soy Broth (BD Difco, Thermo Fisher Scientific) and Luria Bertoni Broth (BD Difco, Thermo Fisher Scientific). Five non-commercial media for halophile/halophilic microorganisms (Rodriguez-Valera et al., 1983; Oren et al., 1995; Payne et al., 2010; Makhdoumi-Kakhki et al., 2012) and one self-designed medium based on the chemical composition of lithium brines (Cubillos et al., 2018) were also used as basal media. 700 μL of concentrated brines at room temperature (25°C) and 7 mL of each medium were added in plastic sterile containers of 15 mL. The enrichment cultures were grown at 37°C (temperature used for the culture of extreme halophiles with salinities of 20 % w/v NaCl or more; Schneegurt, 2012) and agitated at 130 rpm for 4 weeks. The observation of turbidity of media (with respect to a negative control) was considered indicative of microbial growth. Subsequently, cultures were grown in Luria Bertoni Broth (due to positive results) then to 50 mL of medium using 5 mL of the previous culture as inoculum and agitated at 130 rpm for 3 months, until turbidity was displayed. Three transfers of inoculum to fresh medium were performed each week and the colonies obtained were isolated using Luria Bertoni agar at 25°C, reflecting the highest air temperature reported from Salar de Atacama (Alonso and Risacher, 1996) where the brines were obtained. In parallel, the cells were stained using Gram stain to allow their initial characterization. Furthermore, the isolates obtained were evaluated metabolically using the Biolog GEN III MicroPlate^TM^ system (Biolog^TM^, United States) following the manufacturer’s guidelines. The microplates were read by Infinite M200 PRO plate reader, TECAN. Positive reactions were considered greater than 50% of positive control, while negative reactions less than 25% of positive control. The enzymatic profiles of isolates were performed using API^R^ ZYM Galleries (bioMérieux Inc, France), following the manufacturer’s protocol.

### Microbial Growth of LIBR002 and LIBR003 Strains

The microbial growth of LIBR002 and LIBR003 strains was evaluated in Luria Bertoni Broth medium (BD Difco, Thermo Fisher Scientific) under different conditions: Temperature (20–45°C), NaCl concentrations (0–4.2 M), lithium concentrations (0–7.2 M Li^+^), and pH (2–9) at 660 rpm in triplicate. Bacterial growth was evaluated (OD_600__nm_) using Corning^R^ 96 Well Clear Polystyrene Microplates (Life Sciences) and evaluated for 30 h by a Microplate Spectrophotometer (Multiskan^TM^ GO Thermo Scientific, Japan). Furthermore, to determine the inhibitory effects of lithium, cultures were grown in equimolar concentrations of Na/Li (Condition 1: 0.1 M; Condition 2: 0.5 M; Condition 3: 1 M) or with variation of Na/Li ions concentrations (Condition 4: 0.5 M Na-0.1 M Li or Condition 5: 0.1 M Na-0.5 M Li).

### DNA Extraction and Sequencing

DNA extraction was conducted from a single colony using the GeneJET genomic DNA Purification Kit (Thermo Fisher Scientific, United States), following the manufacturer’s protocol. DNA concentrations and quality were measured by Nanodrop spectrophotometer (Nanodrop 8000, Thermo Fisher Scientific, Ottawa, ON, Canada). The oligonucleotide primers Eub27F (5′-AGAGTTTGATCMTGGCTCAG-3′) and Eub1542R (5′-AGAAAGGAGGTGATCCAGCC-3′) (Stackebrandt and Liesack, 1993) were used to amplify 16S rRNA gene fragments using 30–50 ng of gDNA as template. The PCR reaction was performed in a final volume of 25 μL, containing 5 μL PCR GoTaq Buffer 5×, 1.7 μL MgCl_2_ (1.7 mM), 1 μL dNTPS (2 Mm final), 1 μL of each primer (0.8 μM), 0.25 μL GoTaq DNA polymerase (1.25 U) and 2 μL of DNA. The amplicon products were visualized on agarose gel at 0.8% and were selected for its sequencing. Full-length bacterial 16S rRNA gene sequences of approximately 1,400 bp were sequenced using Sanger sequencing technologies (Macrogen Inc., South Korea). These complete sequences of the 16S rRNA gene of isolates have been deposited in GenBank^1^ under access number MK421396 (isolate LIBR002) and MK421402 (isolate LIBR003).

### Classification of Halophilic and Halotolerant Microorganisms

We undertook a detailed bibliographical review to determine validly described species associated with NaCl belonging to the genus *Bacillus*. Halophile or halotolerant type strains and their sequences were obtained by the List of Prokaryotic Names with position in the Nomenclature (LPSN) and used for phylogenetic analysis. The requirements of NaCl (halophiles) or degree of tolerance (halotolerants) were verified with the concentrations of salts reported in each publication for optimal growth or maximum growth obtained. In the case of *Bacillus*-tolerant strains, they were subcategorized according to Larsen’ Classification (Larsen, 1986) as slightly or moderately halotolerant. For halophiles, they were subcategorized as slightly or moderately halophile according to Kushner’ classification (Kushner, 1978). Sequences of *Bacillus*-tolerant isolates were obtained from GenBank (see text footnote 1), according to each access number (Rodríguez-Contreras et al., 2013; Martínez et al., 2018).

### Phylogenetic Analysis

The 16S rRNA sequences were analyzed and assembled by ChromasPRO software (version 2.1.8). Sequences were compared with GenBank using Blastn and Ribosomal Database Project (RDP). Furthermore, the complete 16S rRNA gene sequences (∼1,400 bp) of isolates were aligned using Muscle (Edgar, 2004). A phylogenetic reconstruction was generated and analyzed by maximum parsimony analysis (evolutionary changes) using a bootstrap of 1,000 interactions and the Neighbor-Joining algorithm with the Jukes-Cantor substitution model in Mega 7 (Kumar et al., 2016).

### Preparation of Fatty-Acid Methyl Esters (FAMEs)

The estimation of fatty acids was performed via the extraction to FAMEs (Komagata and Susuki, 1987) and MIDI protocol (Sasser, 1987). Each sample was subjected to saponification by adding 1 mL of 3.75 M NaOH solution in MeOH/H_2_O (1:1 v/v) to a glass tube containing 6 mg of lyophilized mass of strains. The tubes were vortexed, sealed with Teflon, and heated at 100°C for 5 min. Samples were then vortexed vigorously for 10 seconds and returned to the water bath for heating by 30 min. The samples were cooled firstly, and then methylation step was initiated by the addition of 2 mL of 6.0 M HCl solution in MeOH/H_2_O (1:2 v/v). The samples were again sealed with fresh Teflon prior to capping, vortexed and then heated at 80°C for 10 min. The samples were cooled to room temperature, and 1.25 mL of n-hexane/methyl-tert-butyl ether (1:1 v/v) was added and gently shaken on a rotator for about 10 min. The tubes are uncovered and the aqueous phase (bottom) pipetted and discarded. The organic layer containing the methylated fatty acids was washed using 3 mL of 0.3 M NaOH solution, tubes then recapped and stirred for 5 min. After uncapping, the upper n-hexane/methyl-tert-butyl ether phase containing the FAMEs was transferred to vials sealed for analysis by GC.

### Analysis and Quantification of FAMEs

The quantification of fatty acid methyl ester derivatives was performed at the Universidad de Antofagasta, Chile using a gas chromatograph (Shimadzu GC-2010, Shimadzu Co, Kyoto, Japan) equipped with a split/splitless capillary injector and flame ionization detector (FID) locked to a capillary column: 30 m in length, internal dimension 0.32 mm with a 0.25 μM film thickness, coated with 90% biscyanopropyl and 10% phenyl cyanopropyl polysiloxane, RT2330 RESTEK, United States. The temperature of the split injector was 250°C. High-purity grade Nitrogen (N_2_) was used as the carrier gas at a flow rate of 11.25 mL/min. The FID flame was produced using synthetic air and high-purity grade hydrogen. The temperature of the detector was 280°C. Quantification of FAMEs was achieved by comparing the retention times of the methyl ester derivatives obtained from external standard FAME-Mix (FAME Mix C_4_-C_24_, Supelco Analytical, Sigma-Aldrich).

### Statistical Analysis

The generation time (g) was calculated by equation *g* = *t*/*n*, where t was time (hours) and n as number of generation (Madigan et al., 2008). The generation time obtained under each growth condition were compared through one-way analysis of variance (ANOVA), followed by Bonferroni’s *post hoc* test. Significant differences are represented with ^*^p < 0.05, ^∗∗^*p* < 0.001, and ^∗∗∗^*p* < 0.0001. Figures were generated using GraphPad Prism, version 6.0 (GraphPad Software, Inc., La Jolla, CA, United States).

### Scanning Electron Microscopy (SEM)

Cells in suspension were fixed in 2% glutaraldehyde and 2% paraformaldehyde in 0.1 M sodium cacodylate buffer pH 7.2 and kept in at 5°C until further processing. After this, cells were washed for 3 × 5 min with buffer and dehydrated in an ethanol gradient – 30, 50, 70, 80, 90, and 95%, 2 × 100% v/v- 10 min per step and then transferred onto a 0.22 μm polycarbonate filter using a mild vacuum. The filter was kept in 100% ethanol then incubated for 1 min in HMDS (hexamethyldisilazane) before air-drying the filter. Pieces of the filter were mounted onto an aluminum sample pin using carbon conductive tabs. Samples were sputter coated with 10 nm gold/palladium (80/20). Samples were then imaged using a JEOL JSM 6390 LV Scanning Electron Microscope operated at 5 kV in the Living Systems Institute, at the University of Exeter, United Kingdom.

## Results

### Halophilic *Bacillus* Extend Beyond NaCl to LiCl

**FIGURE 2:**
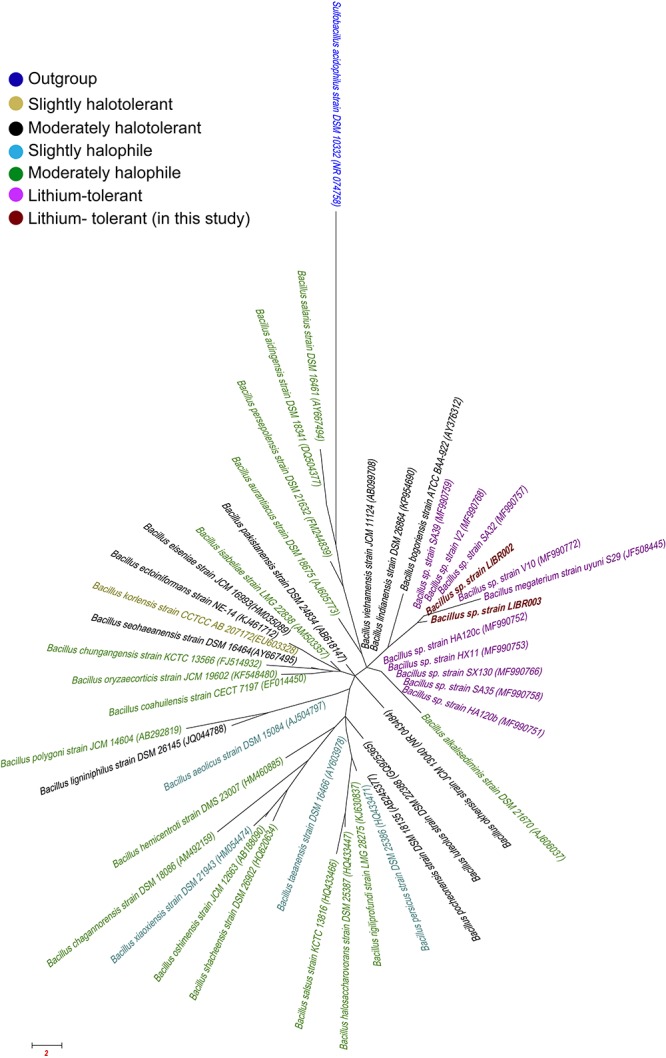
Phylogenetic tree using sequence of 16S rRNA gene (∼1400 bp) of *Bacillus* related-NaCl. The strains obtained in this study are shown in dark red, lithium-tolerant in pink, slightly halotolerant in olive, moderately halotolerant in black, slightly halophile in teal and moderately halophiles in green color. Access numbers are shown in parentheses and *Sulfobacillus acidophilus* was used as outgroup (blue).

**FIGURE 3:**
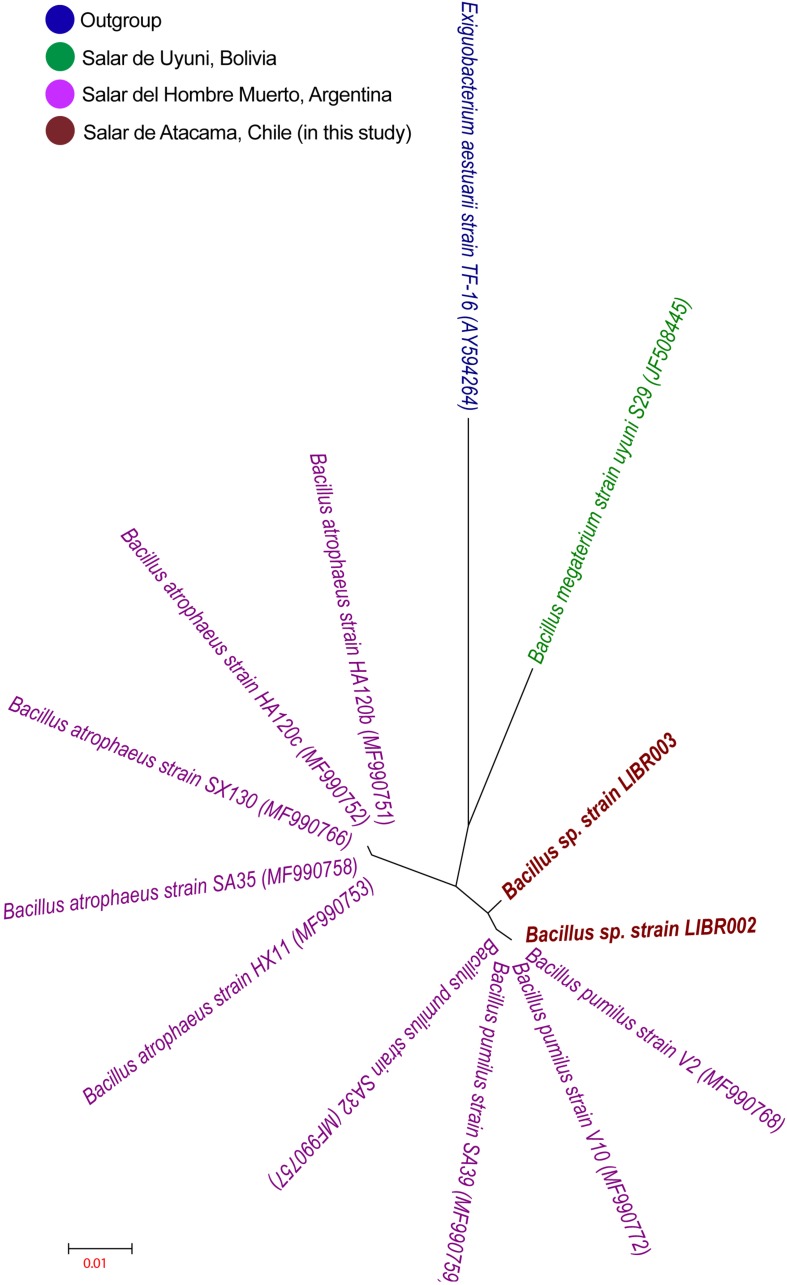
Phylogenetic tree using sequence of 16S rRNA gene of *Bacillus* isolated from the Lithium Triangle Zone. The strains obtained in this study are shown in dark red and the access number is shown in parentheses. The values of bootstrap >50% are shown (1,000 interactions). *Exiguobacterium aestuarii* was used as outgroup.

The liquid culture of lithium brines, using Luria Bertoni Broth medium, showed turbidity after 3 months, unlike the rest of the other media. Three colonies in total were obtained from enrichment cultures, though two exhibited different colony morphology, then they were isolated and were designated as LIBR002 and LIBR003 strains. Both isolates were classified as Bacteria and as members of the *Bacillus* genus. The phylogeny of NaCl-associated *Bacillus* (Figure 2) showed that the isolates obtained in this study (in dark red) grouped close to lithium-tolerant isolates from Salar del Hombre Muerto and Salar de Uyuni and apart from halophilic and halotolerant *Bacillus*-species. Partial 16S rRNA gene sequences (∼1,400 bp) obtained from isolates were compared with sequences from *Bacillus* obtained from the Lithium Triangle Zone (Figure 3). Strain LIBR002 was phylogenetically related with *Bacillus pumilus strain V2* (from Salar de Hombre Muerto), while strain LIBR003 showed low similarity with other sequences. Of all the type strain sequences (44) analyzed from NaCl-associated *Bacillus*, 48% were halophiles (moderately and slightly), 27 % were halotolerant and 25 % were lithium-tolerant.

**FIGURE 4:**
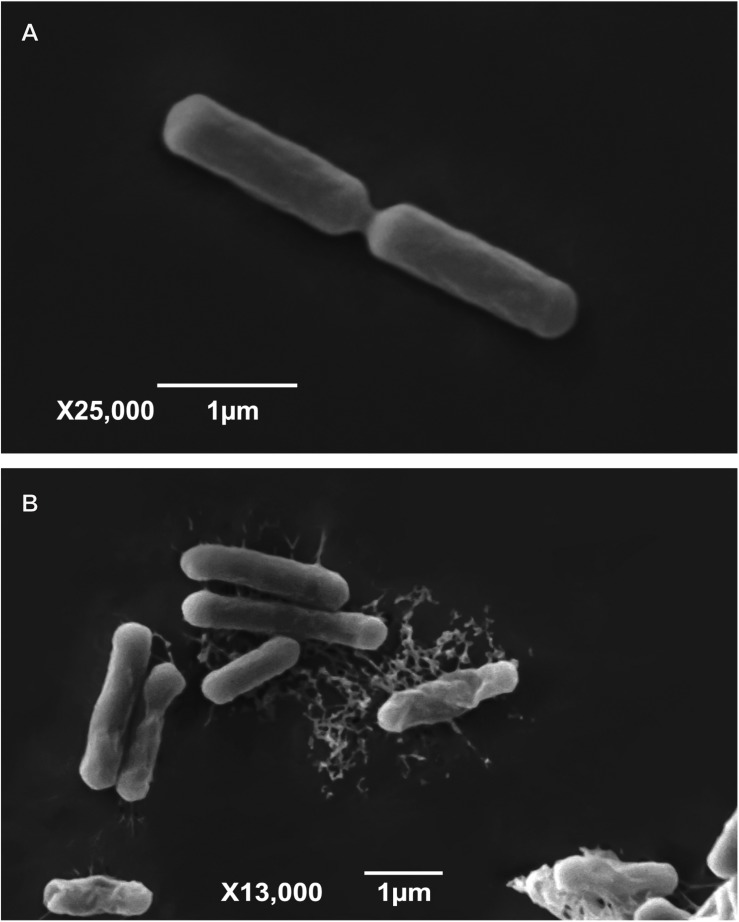
Visualization of *Bacillus* sp., strains from lithium brines by Scanning Electron Microscopy (SEM). **(A)** LIBR002 strain; **(B)** LIBR003 strain.

According to the first hit of Blastn (NCBI), strain LIBR002 showed 99% 16S rRNA similarity with *Bacillus safensis* FO-36b and strain *LIBR003* with *Bacillus altitudinis* strain P-10 (100% similarity). The isolates were classified as Gram-positive and their colony morphology showed clear differences. Strain LIBR002 was irregular, with a dry surface and dark brown color, which expanded through agar after 1 week and LIBR003 was circular, with an undulate edge, brilliant surface, and beige color. Further, the cellular morphology of both isolates was classified as round-ended cylinders (Figure 4). Additionally, the microbial cells of LIBR003 strain were linked by a matrix (Figure 4B), in contrast to LIBR002 cells (Figure 4A), which were unconnected.

### Salinity Effects on Microbial Growth

**FIGURE 5:**
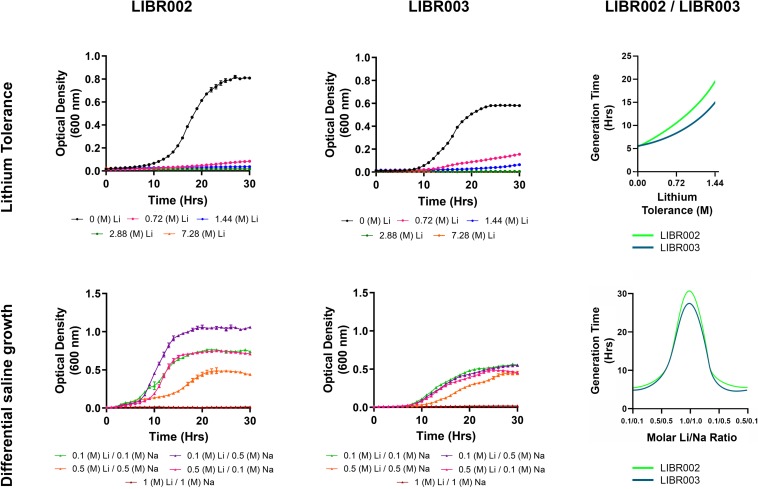
Microbial growth of isolates LIBR002 and LIBR003 indifferent LiCl and NaCl concentrations. Values are mean ± standard error of the mean of experiments (SEM). The trend lines are represented as solid lines.

The presence of lithium in the culture medium had an important effect on the growth of bacterial cells. On average, the presence of lithium (0.72 M Li^+^) doubled the generation time of strain LIBR002, and increased generation times by 1.5 times in strain LIBR003 (Figure 5), compared to lithium-free conditions. When lithium concentrations were increased to 1.44 M, generation times increased 3.6 and 2.7 times in strains LIBR002 and LIBR003, respectively. Under the highest lithium concentrations (2.88 and 7.2 M), no growth was observed in either of the strains. Although both isolates were able to grow in presence of lithium, they were also able to growth without this cation (0 M Li).

In our experimental assessment of the effects of differential saline conditions on microbial growth, both isolates showed the lowest generation time under condition 4 (Figure 5). Later, the generation time of strain LIBR002 were increased by minutes for conditions 5, and 1, and increased by 1 h under condition 2. Conversely, in strain LIBR003, the generation time increased by at least 1 h under conditions 1, 2, and 5. Therefore, growth was worse under condition 3, where lithium/sodium concentrations were highest, increasing the generation time by 7 for strain LIBR002, and by 5.8 times for strain LIBR003 when compared to optimum growth.

Both isolates were able to grow at temperatures between 20°C and 45°C (Supplementary Figures S1, S2 and Supplementary Table S1). However, for both strains, optimum growth was attained at 37°C, and growth was lowest at 20°C. There was no evidence that either strains required NaCl for growth (4.65 for LIBR002 and 5.34 h for LIBR003) (Supplementary Figures S1, S2 and Supplementary Table S2). However, strain LIBR002 showed the lowest generation time at a NaCl concentration of 0.34 M, and LIBR003 at 0.17 M NaCl. Furthermore, the isolates studied here could be classified as slightly tolerant strains, according to the halotolerant classification (Larsen, 1986), as growth was apparent at 0 M NaCl. Neither isolate grew at pH 2, but growth was observed at pH 3 and 4 in the LIBR002 strain (Supplementary Figures S1, S2 and Supplementary Table S3). Optimal growth was observed at pH 6 for strain LIBR002, and pH 5 for strain LIBR003 – hence they can be classified as either neutrophile (Madigan et al., 2008) and moderately acidophile (Johnson, 2008), respectively.

### Fatty Acid Profiles

Five different fatty acids were identified in each bacterial strain (Table 1). Myristoleic acid was the most abundant in both strains (LIBR002 = 16.87 ± 6.7 ppm and LIBR003 = 15.36 ± 3.2 ppm). The minor fatty acids concentrations recorded from LIBR002 included palmitoleic (6.05 ± 2.0 ppm), palmitic (2.34 ± 1.6 ppm), oleic (1.72 ± 0.5 ppm), and myristic acid (1.36 ± 1.3 ppm). Fatty acids found in lower concentrations in LIBR003 included palmitic (4.45 ± 1.7 ppm), margaric (3.53 ± 1.3 ppm), palmitoleic (2.52 ± 0.9 ppm), and pentadecanoic acid (1.98 ± 0.7 ppm). Margaric and pentadecanoic acids were not present in LIBR002, and myristic and oleic acids were not recorded from LIBR003.

**TABLE 1:** Fatty acid composition of species of *Bacillus* of lithium brines.

				**Strain**	
				**LIBR002**	**LIBR003**
				
**Retention Time (min)**	**Fatty acid**	**Compound common name**	**Compound systematic name**	**Concentration (ppm)**	
17.08	C14:0	Myristic acid	Tetradecanoic acid	1.36 ± 1.3	ND
18.48	C14:1 (C9)	Myristoleic acid	*cis*-9-tetradecenoic acid	16.87 ± 6.7	15.36 ± 3.2
19.12	C15:0	Pentadecanoic acid	Pentadecylic acid	ND	1.98 ± 0.7
21.04	C16:0	Palmitic acid	Hexadecanoic acid	2.34 ± 1.6	4.45 ± 1.7
22.05	C16:1 (C9)	Palmitoleic acid	*cis*-9-hexadecenoic acid	6.05 ± 2.0	2.52 ± 0.9
23.04	C17:0	Margaric acid	Heptadecanoic acid	ND	3.53 ± 1.3
26.35	C18:1 (C9)	Oleic acid	*cis*-9-octadecenoic acid	1.72 ± 0.5	ND

*ND, not detected.*

### Metabolic and Enzymatic Characterization of Isolates

The isolates from lithium concentrated brines showed metabolic differences according to Biolog GEN III MicroPlate^TM^ comparisons (See Supplementary Table S4). Both isolates were able to use a range of substrates as carbon sources: Citric Acid, L-Malic Acid, D-Trehalose, D- Cellobiose, β-Methyl-D-Glucoside, D-Salicin, α-D-glucose, D-mannitol, L-Aspartic Acid, L-Glumamic Acid and Pectin. Strain LIBR002 could use the following as carbon sources: L-Lactic Acid, Sucrose, D-Mannose, D-Fructose, D-Sorbitol, Glycerol, D-Turanose, D-Galacturonic Acid, D-Gluconic Acid and L-Galactonic Acid Lactone. Carbon sources specific to strain LIBR003 included D-Aspartic Acid, Quinic Acid, Gentibiose, Methyl Piruvate, γ-Amino-Butryric Acid, L-Serine, Bromo Succinic Acid. The chemical sensitivity of these strains is detailed in Supplementary Table S4.

Both isolates show the following enzyme activity Enterase, Enterase-Lipase, Valine arylamidase and Naphthol-AS-BI-phosphohydrolase (Supplementary Table S5). The LIBR002 strain displayed specific metabolic machinery including Leucine arylamidase, Cysteine arylamidase, Acid phosphatase, β-glucosidase and N-acetyl-β-glucosaminidase, while the LIBR003 strain showed metabolic activity with α-chymotrypsin and Trypsin.

## Discussion

Industrial lithium brines from Salar de Atacama (Chile) can be considered as an extreme and unusual saline environment (total salinity 556 g/L; 11.7 M LiCl), and we report the isolation of two lithium tolerant (able to grow up to 1.44 M Li) *Bacillus* strains from these brines. Saline (i.e., NaCl-associated) environments are typically considered as restrictive, where only halotolerant and halophile taxa that due to specific adaptations can counter the presence of various ions, and live (Zahran, 1997). Such microorganisms are often classified according to their NaCl requirements for growth (Kushner, 1978; Tiquia et al., 2007). Conversely, organisms able to grow in high lithium concentrations are classified as lithium-tolerant. A limited group of bacteria are known to be tolerant of high lithium concentrations (up to 1 M Li): *Micrococcus varians* ssp. *Halophilus* (1.5 M Li; Kamekura and Onishi, 1982), *Rhodococcus* sp., A5wh (1 M Li; Belfiore et al., 2018), *Staphylococcus sciuri* strain LCHXa (1 M Li; Vilo et al., 2019) and *Bacillus-* species (1.42 M; Martínez et al., 2018). According to current knowledge, lithium does not play a vital function in microbial cells (Aral and Vecchio-Sadus, 2008), however, it has been reported an inhibitor effect in lactic acid bacteria (Lapierre et al., 1992). We showed that lithium has considerable effect on isolates, decreasing growth and increasing generation time at lower lithium concentrations (0.72 M Li). Bacteriostatic effects, such as inhibition of growth (Nemeth et al., 2015) in lithium-tolerant strains (free-living bacteria), has not been examined in depth, although it is apparent that direct contact in liquid media increases the sensitivity of tolerant microorganisms, even more than that seen with solid media (Majzlik et al., 2011; Martínez et al., 2018; Vilo et al., 2019). LiCl as well as MgCl_2_ are classified as chaotropic solutes that reduce the electrostatic interaction between water molecules and biological macromolecules (by hydrogen bonding) (Cray et al., 2013), resulting in macromolecule destabilization (e.g., proteins) through the denaturation of tertiary and quaternary structure, triggering the inhibition of microbial growth (Hallsworth et al., 2003; Williams and Hallsworth, 2009; Cray et al., 2013). This thermodynamically non-favorable effect has been reported in *Pseudomonas putida* at 0.5 M of LiCl, a concentration that inhibited 50% of growth (Hallsworth et al., 2003) – similar to the growth of our *Bacillus*-cultures exposed to minimum lithium concentrations (0.72 M). Genomic expression in the presence of chaotropic solutes includes the up-regulation of proteins involved in the stabilization of proteins in thermodynamically non-favorable conditions (e.g., HtpG, DnaK, and GroEL) (Hallsworth et al., 2003), and overexpression has also been reported in lithium-tolerant bacteria such as *Rhodococcus* sp., (Belfiore et al., 2018). Additionally, the high concentration of lithium in concentrated brines (11.7 M LiCl; Cubillos et al., 2018) could explain the extended time required (months) to obtain viable-cultures, as has been previously described for obtaining successful cultures from a brine lake with high concentrations of MgCl_2_ (Hallsworth et al., 2007). Experimental studies have shown that many extremely halophilic microorganisms can grow in NaCl-saturated solutions up to 5 M NaCl (a_w_ = 0.755), and that a small number can replicate at lower water activities in MgCl_2_-rich brines (Stevenson et al., 2015; Lee et al., 2018). Whereas we did not measure the water activity of lithium-concentrated brines (Cubillos et al., 2018), we believe that the 11.7 M Li^+^ brine would also exhibit a water activity considerably below 0.755.

The phylogeny of NaCl-associated *Bacillus* – (halophile, moderately halophile, and halotolerant groups) showed that lithium-tolerance isolates are located in two monophyletic groups, apart from NaCl-associated organisms (Figure 2). To date, all lithium-tolerant isolates obtained from the Lithium Triangle Zone are reported as also being NaCl-tolerant (Rodríguez-Contreras et al., 2013; Martínez et al., 2018) and could be related phylogenetically (Figure 3). This phylogenetic separation between the lithium-associated taxa from the other NaCl-associated taxa may indicate that NaCl-tolerance could allow the survival of these microorganisms in habitats with other saline stressors, including lithium brines, with respect to halophile, moderately halophile and halotolerant organisms. It has been reported that some halotolerant organisms, including *Bacillus* show increased content of acidic amino acids in surface proteins in the absence or presence of NaCl, in contrast to surface proteins of thermophiles which display abundant acid and basic amino acid residues (Fukuchi et al., 2003; Martínez et al., 2018).

The maximum concentrations of NaCl reported to support growth of *Bacillus*-spp to date is 20% salinity (Lim et al., 2006). Here, we show that this microbial group can also grow in at high concentrations of salts other than NaCl, namely LiCl. Although we showed an increase in generation time in the isolates in the presence of lithium (in comparison without lithium), the substitution of salts could help the survival of microorganisms, as reported from the growth of halophiles (Kamekura and Onishi, 1982). Generation time decreased considerably in cells grown in balanced concentrations of sodium/lithium ions (Na^+^/Li^+^; T_gLIBR002_ = 4.34 ± 0.02 and T_gLIBR003_ = 4.73 ± 0.02 h), and cultures were only grown in the presence of lithium (Li^+^; T_gLIBR002_ = 10.68 ± 0.45 and T_gLIBR003_ = 8.27 ± 0.21 h). Nevertheless, when both cations had concentrations >1 M, generation time increased considerably (Na^+^/Li^+^; T_gLIBR002_ = 30.87 ± 7.76 and T_gLIBR003_ = 27.61 ± 0.67 h). This increase of generation time could reflect that at low lithium concentrations, this cation could accumulate intracellularly, whereas at high NaCl concentration (3 M), accumulation is reduced (Tanjasiri et al., 2013). This balance has also been reported to counter biological stress associated with chaotropic, through the use of kosmotropic solutes (such as NaCl) (Hallsworth et al., 2003; Cray et al., 2013; Rubin et al., 2017). Consequently, we suggest that the inhibition effects of lithium is not exclusive to lactic microorganisms, but can be countered by a balance of sodium cations in the culture medium, though its growth will be lower than that shown in cells under optimal growth conditions (Williams and Hallsworth, 2009; Cray et al., 2015).

The fatty acid composition present in cellular membranes is a useful tool for bacterial systematic classification (Komagata and Susuki, 1987), as a viability marker (Sephton et al., 2013) and to determine microbial diversity in hypersaline environments (Ventosa and Ventosa, 2004). The fatty acid profiles of both isolates showed high concentration of membrane unsaturated fatty acids (LIBR002 = 87%; LIBR003 = 64%). Saturated (LIBR002 = 7:1; LIBR003 = 2:1) fatty acids would likely be crucial as a survival mechanism to counter lithium stress. The degree of unsaturated fatty acids and fluidity decrease (to gel state) in extremophile membranes have both been considered as adaptation mechanisms under high hydrostatic pressure (DeLong and Yayanos, 1985), high and low temperature (Koynova et al., 1997; Sakamoto and Murata, 2002) and high salinity (Turk et al., 2003). Furthermore the fatty acids reported from the *Bacillus* genus have been classified as six branched (anteiso-C_15_, anteiso-C_17_, iso-C_14_, iso-C_16_, and iso-C_17_) and normal (nC_14_ – n-C_16_) (Kaneda, 1967), where the saturated fatty acids are shared with our isolates (Table 1). Unsaturated fatty acids reported from *Bacillus* membranes include *cis*5, *cis*8, *cis*10, and *cis*9 (Kaneda, 1977), where the latter being more abundant in our isolates (Table 1). An increase in unsaturated fatty acids (Heipieper et al., 1992; Hallsworth et al., 2003) and the reduction of membrane permeability in non-lithium tolerant *Pseudomonas aeruginosa* by downregulation of the outer membrane protein F have been reported as mechanisms to counter chaotropic effects (Yoshimura and Nikaido, 1982; Hallsworth et al., 2003). In consequence, chaotropic effects, high viscosity (26 times more than water; Cubillos et al., 2018), high salinity (556 g/L) and the high lithium concentrations (11.7 M LiCl) present in brines likely limit the optimal microbial growth of bacteria inhabiting this unusual environment. Nevertheless, the fluidity change of cell membranes could aid survival allowing a niche for microbial life and growth.

Both isolates were classified as *Bacillus*-like organisms and slightly halotolerant. Despite considerable heterogeneity seen in the 16S rRNA gene of *Bacillus* genus (Ash et al., 1991), Firmicutes was one of the phylum dominant in the concentrated brines (Cubillos et al., 2018). Further, according to the Kaneda classification (Kaneda, 1977), both isolates have a high proportion of unsaturated FAs, therefore can be classified in a third group with psychrotolerant species (Diomandé et al., 2015). It is important to mention that these fatty acid profiles are, to our knowledge, the first microbial description of lithium-associated *Bacillus* reported to date.

Salar de Atacama and its brines, especially concentrated brines, can be considered as poly-extreme environments, with conditions generally considered unfavorable for life, i.e., presence of chaotropic solute as main salt LiCl (11.7 M), high salinity (556 g/L), high evaporation rates (3,700 mm/year), high solar radiation (6.3 × 10^6^ cal/ m^2^ day) and very low average precipitation rates (10 mm/year) (Alonso and Risacher, 1996; Garcés, 1998; Garret, 2004; Cubillos et al., 2018). As such, as expected we only obtained spore-forming bacteria (*Bacillus*). This reflects the fact that endospore-forming Firmicutes are favored in habitats with multiple environmental limiting factors (Filippidou et al., 2016), and that these organisms generate spores in response to saline-stress (Garabito et al., 1998). Spores are recognized as the hardiest form of life on Earth reported to date, and represent a highly successful strategy for the survival and dispersal of microbial life (Nicholson et al., 2000). Some members of the *Bacillus* genus are widely known as spore-forming bacteria (Nicholson et al., 2000) with spores displaying cryptobiosis over extremely long durations (Gest and Mandelstam, 1987). This has permitted the isolation of *Bacillus* from ancient sources including amber (Greenblatt et al., 1999), salt deposits (Dombrowski, 1963), and 250 million-year-old salt crystals (Vreeland et al., 2000). As such, we suggest that spore-production represents an adaptation mechanism allowing survival under saline-stress that extends beyond NaCl: furthermore, lithium can produce a semi-vegetative state in *Bacillus*, as reported in the *Halobacteriaceae* (a markedly reduced ATP content) (Nicholson et al., 2000), as well as a dormant state described from environments with high concentrations of chaotropic solutes such as MgCl_2_ (Hallsworth et al., 2007). Additionally, as bacterial abundance is usually negatively correlated with total salts in a given environment (Ragab, 1993), this likely explains the low cellular abundance we observed in lithium concentrated-brines (data not shown).

In some environments, the presence of extreme physical parameters lead to inhospitable conditions for life, including oxidizing agents, desiccation, elevated ultraviolet, and γ-radiation: *Bacillus* spores however, are characteristically tolerant to such conditions (Link et al., 2004; Crawford, 2005). This capacity has led to *Bacillus* spores to be included as models for the study of interplanetary transfer of microorganisms (Nicholson and Schuerger, 2005). The habitability of planets or extra-terrestrial environments is typically restricted to the presence of liquid water (Marion et al., 2003), extending potential habitats to brines or salty oceans on Mars (Benison and Bowen, 2006), Europa (Marion et al., 2003; Hand and Carlson, 2015) and Enceladus (Thomas et al., 2016). However, chaotropicity in soils or brines from these celestial bodies has to be considered. Chaotropic solutes can modify microbial diversity e.g., in brines of Salar de Uyuni (MgCl2; Rubin et al., 2017) and Salar de Atacama (LiCl; Cubillos et al., 2018), and could even limit microbial life by reducing the water activity and destabilization of macromolecules (Hallsworth et al., 2003, 2007; Williams and Hallsworth, 2009).

Temperature, chaotropicity, and lower water activity, among other parameters could be used to define the biophysical limits of microbial life on Earth (Lee et al., 2018). Nevertheless, the chaotropicity of brines has been considered one of the most crucial parameters affecting the development of life on Earth (Williams and Hallsworth, 2009; Rubin et al., 2017). In this context, the search for microbial life on Mars should be focused on: (1) halophile or halotolerant organisms from brines extending beyond NaCl, given that Martian-brines may exhibit different chemical compositions to terrestrial brines affecting their habitability (Fox-Powell et al., 2016) and (2) microorganisms that exhibit chaotolerant or chaophilic characteristics to resist under chaotropic conditions (Williams and Hallsworth, 2009; Lee et al., 2018). In conclusion, the habitability of an hypothetic extreme-ecosystem (extra and/or terrestrial) must be evaluated from a viewpoint that includes biological (microbial diversity or isolates), chemical-physical (salinity, temperature, pH, among others), and biophysical (water activity, chaotropicity, and ionic strength) parameters (Stevenson et al., 2015; Lee et al., 2018) that potentially could permit the germination of spores and subsequent growth of well-adapted cells, as reported here.

### Ecology of Lithium Triangle Zone Salares

The majority of halotolerant strains used in phylogenetic analysis of this study were isolated from saline sources (79%). Nevertheless, the remaining strains were isolated from a wide range of habitats including ginseng fields, the intestinal tract of animals and sand. Our lithium-tolerant *Bacillus* strains were obtained from concentrated lithium brines following an industrial process – in order to determine their natural origin, it is necessary to consider the dispersion of endospores. It is not clear from the literature whether obtaining *Bacillus*-strains reflects their presence in a given environment or their elevated capacity for dispersion and adaptation by spores to shift from a state of dormancy to activity, under favorable conditions (Alcaraz et al., 2010). Nevertheless, the transport of spores from one location (i.e., isolation source) to another as product of dust or winds should not be discounted. The feathers of birds like pelicans (Kemp et al., 2018) or flamingos (Yim et al., 2015) could also be considered as mechanical carriers (also to microbial vectors) to transport microbial cells from different habitats. In the Puna zone, where Salar de Atacama, Salar de Hombre Muerto, and Salar de Uyuni are located, three flamingo species are abundant, *Phoenicopterus chilensis* (Chilean), *P. jamensis* (James’s) and *P. andinus* (Andean) (Hurlbert and Chang, 1983). It has been reported that large-bodied taxa (as flamingos) can alter bacterial communities, through dispersion or modifying nutrient concentrations (Jude, 2018). Further, the exclusion of flamingos through industrial activities such as mining can produce shifts in the distribution and abundance of many taxa including diatoms, amoebas, ciliates, and nematodes (Hurlbert and Chang, 1983).

### Lithium in Early Earth: The Possible-Molecular Fossils of Lithium-Organisms

Lithium has two potential origins: as a product of the Big Bang (13.5 billion years ago) or by nuclear reactions induced by energetic cosmic rays in the interstellar medium (Hernanz, 2015). The origin and early development of the Sun and Earth was from the protosolar nebula (4.55 billion years ago) was violent (Robert, 2002). The exact time that life originated on early Earth it is not yet fully known, although there are suggestions that the first life that appeared 3,500 Ma was a prokaryotic chemoautotrophic and anaerobic bacteria (Cavoise et al., 1983). However, this remains controversial, with some workers suggesting an earlier origin for life (Ushikubo et al., 2008).

The Hadean Eon is Earth’s earliest geological epoch (4,560 Ma). Following impacts from extra-terrestrial bodies and the presence of magma oceans, surface temperatures (226–230°C) allowed the vaporization of oceans, resulting in a dense steam atmosphere (Cavosie et al., 2007). Later, reduced surface temperatures (70°C) and the rise of the oceans, resulted in the Archean (4,600 Ma) (Valley et al., 2002; Cavosie et al., 2005, 2007). The oldest terrestrial materials of Hadean-Archean transition (Sleep, 2010) reported to date, are zircons from Jack Hills (Australia) with an age up 4,000 Ma (Ushikubo et al., 2008). These materials are metasedimentary rocks (ZrSiO4) and their elemental composition provides chemical clues to conditions during this part of Earth’s geological history (Ushikubo et al., 2008). Trace elements include uranium, thorium, hafnium and lithium (Sleep, 2010), with the last potentially being crucial for Earth’s history. The highest lithium concentrations of the oldest zircons (up to 49 ppm), compared to younger zircons (2 ppb Li) suggests that their parent magmas incorporated materials from the Earth surface and interacted with liquid water at low temperature (Valley et al., 2002; Ushikubo et al., 2008), similar to decrease of nitrate in Mars surfaces product of chemistry atmospheric shift (Navarro-González et al., 2018). The Li isotope ratios of these zircons supports the idea of existence of a continental crust and oceans that (given the result of the current study) provided potential habitat for microbial life from 4,300 Ma or even earlier (Ushikubo et al., 2008). Therefore, life may have arose in or around the rocks, instead of relying on photosynthesis at the surface (Gaucher et al., 2010). Conversely, this eon was characterized by a period of frequent impacts by asteroids and meteorites, particularly during a period referred to as the late heavy bombardment (3,800–4,100 Ma). Although these events may have had marked negative effects on prehistoric life, it is likely that thermophilic organisms survived (Sleep, 2010). To determine the habitability of early Earth, biosignatures or molecular fossils must be found (Sleep, 2010). In this context, chemo-fossils were reported from zircons, and were described as colonies of unknown and diminutive organisms (Bell et al., 2015). Such early microbial life would be reliant on the presence of adaptations to permit survival under early extreme environments, including the high lithium concentrations present in zircons. In consequence, it is possible that this first life form was ancestral to current lithium-tolerant microorganisms. Although, this cannot be confirmed here, genomic analysis of modern lithium-tolerant organisms (such as our isolates) could potentially provide key information regarding crucial and ancient process of evolution.

## Conclusion

In conclusion, we report the isolation of two bacterial species from one of the most hypersaline environments described to date on Earth (total salinity = 556 g/L), where lithium chloride is the principal salt. Furthermore, we established the bacteriostatic effects of lithium on the microbial cells and we suggest that the NaCl-tolerance, generation of spores and fluidity change of membrane of *Bacillus* could be keys to survive in this poly-extreme environment, as reported elsewhere (Filippidou et al., 2019). The information detailed here is likely relevant to the search for microbial life in planets and other celestial bodies with surficial salt-lakes or brines that provide a potential niche for microbial life. In consequence, these fragile salar ecosystems (salares), known as the Lithium Triangle Zone, must be protected, due at they are base or support of large food chain from animals (flamingos), going through microscopic eukaryotes (ciliates and amoebas), and microbial life (as archaea and bacteria organisms).

## Data Availability

Publicly available datasets were analyzed in this study. This data can be found here: https://www.ncbi.nlm.nih.gov.

## Author Contributions

CFC prepared the samples for analyses and designed the experiment protocols. CFC, CY, JP, and DV performed the experiment. ES was responsible for the figure production. CFC and AP wrote most of the manuscript. MG and CD contributed with the writing of the manuscript. CD was obtained funding for this study. All authors read and approved the final version of the manuscript.

## Conflict of Interest Statement

The authors declare that the research was conducted in the absence of any commercial or financial relationships that could be construed as a potential conflict of interest.

## Footnotes

1 https://www.ncbi.nlm.nih.gov
