# Supplementary material for: Insights Into the Microbiology of the Chaotropic Brines of Salar de Atacama, Chile
Source: Front Microbiol. 2019 Jul 11;10:1611. doi: 10.3389/fmicb.2019.01611 (PMC6637823; doi:10.3389/fmicb.2019.01611)
Supplement: Supplementary file 1 [file Data_Sheet_1.docx]

Supplementary Material

# Supplementary Figures

A)


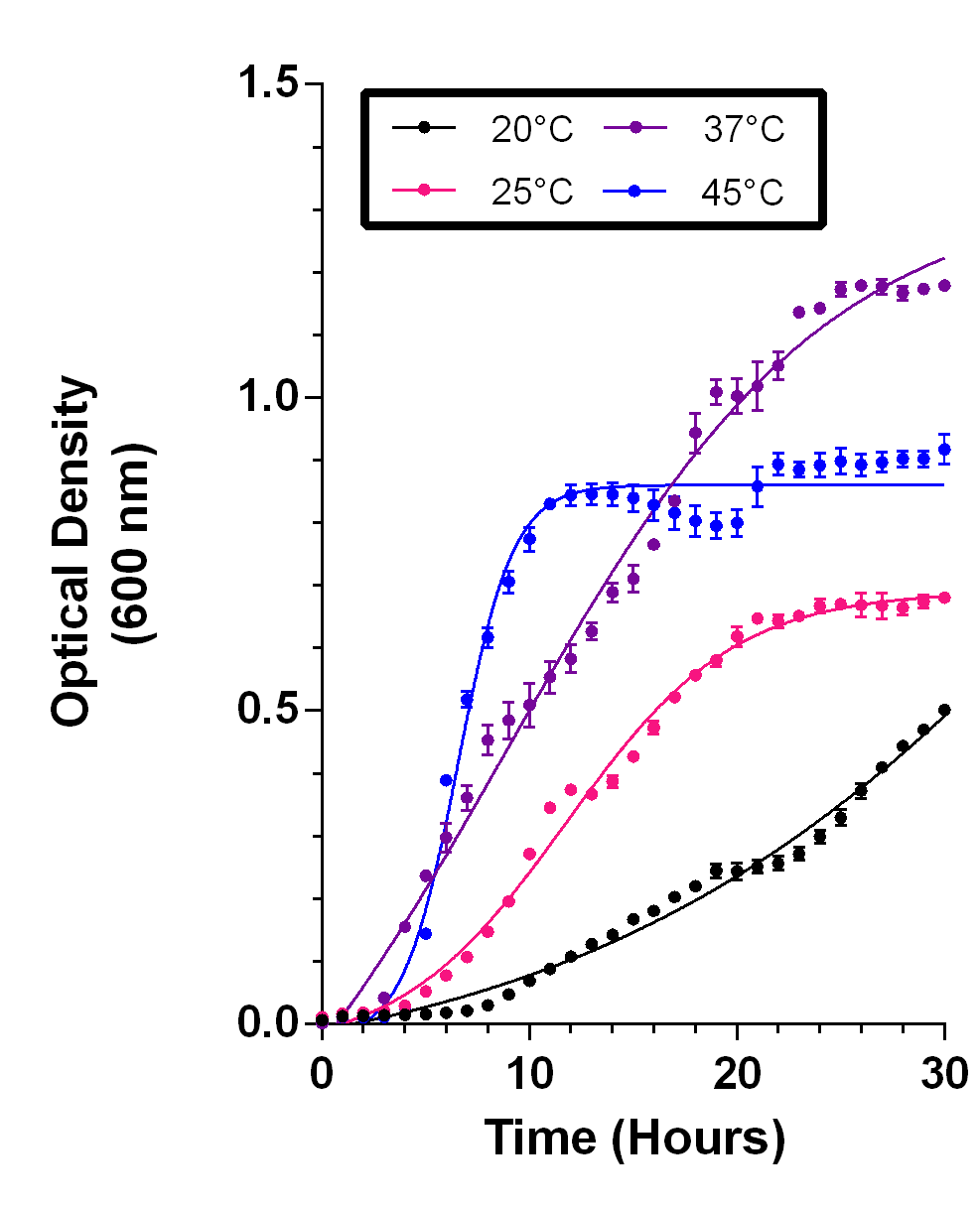


B)


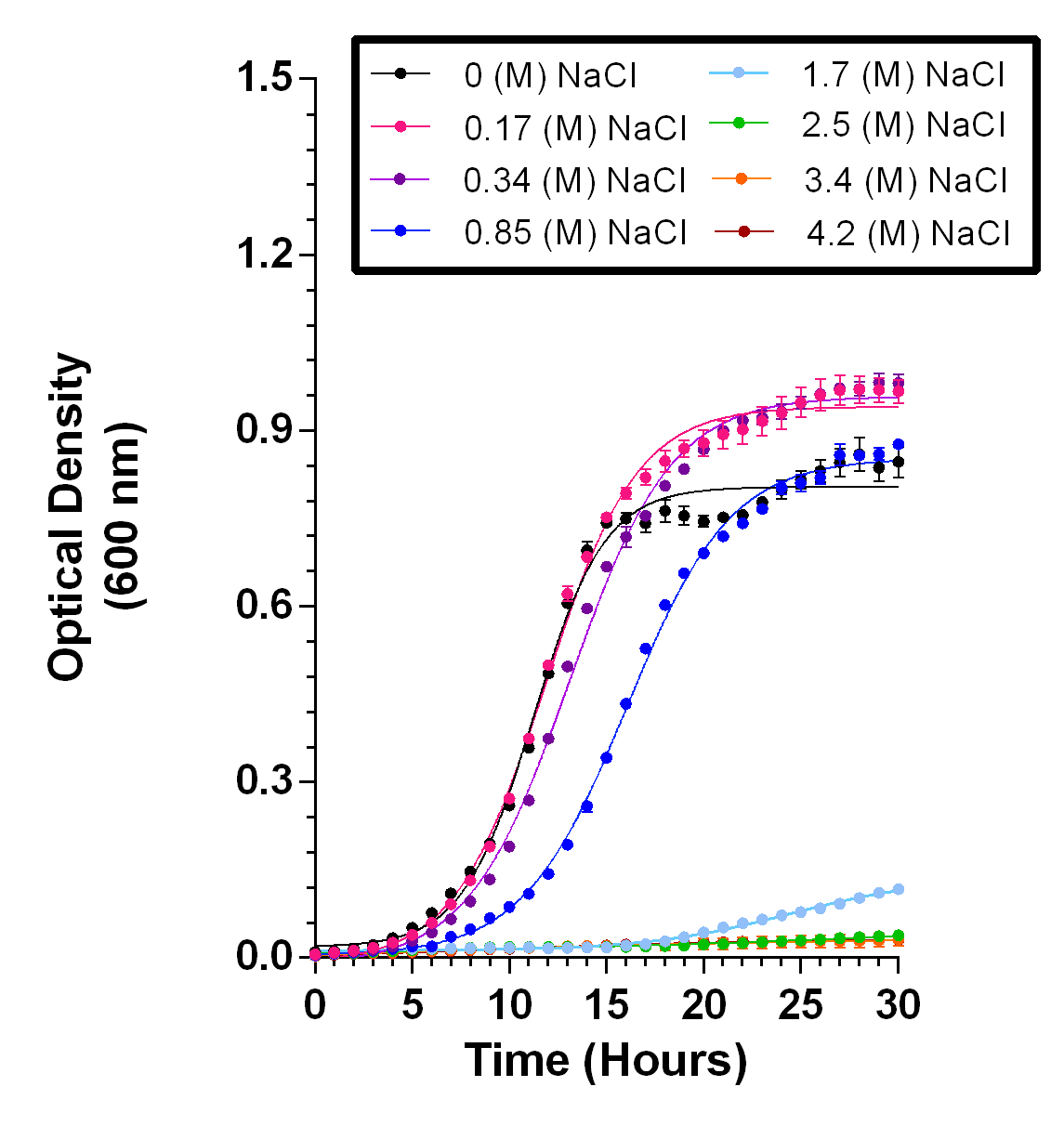


C)


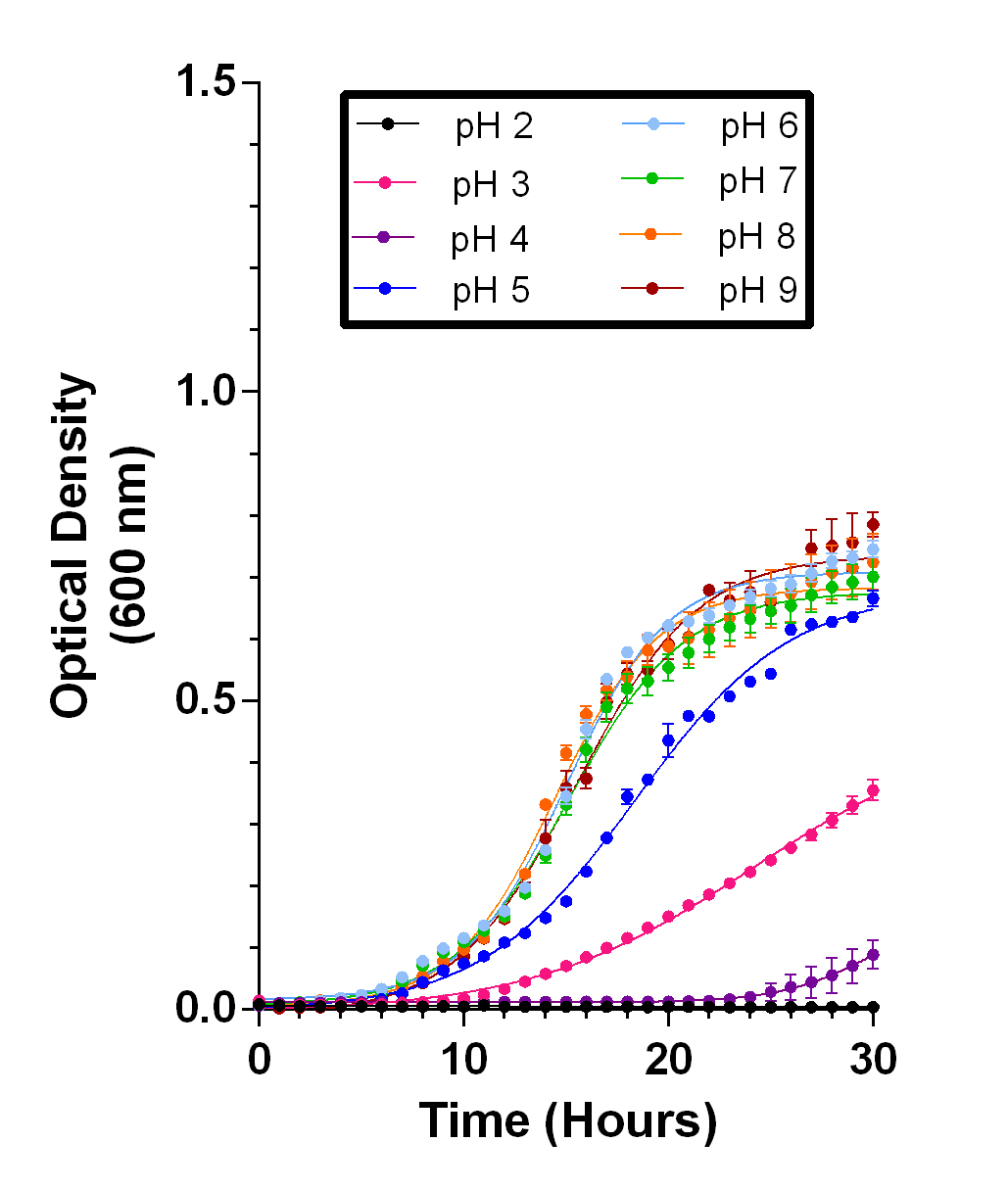


**Supplementary Figure S1**. Microbial growth of LIBR002 strain under different experimental conditions: A) Temperature, B) NaCl- growth and C) pH. Values are mean ± standard error of the mean of experiments (SEM). The trend lines are represented as solid lines.

A)


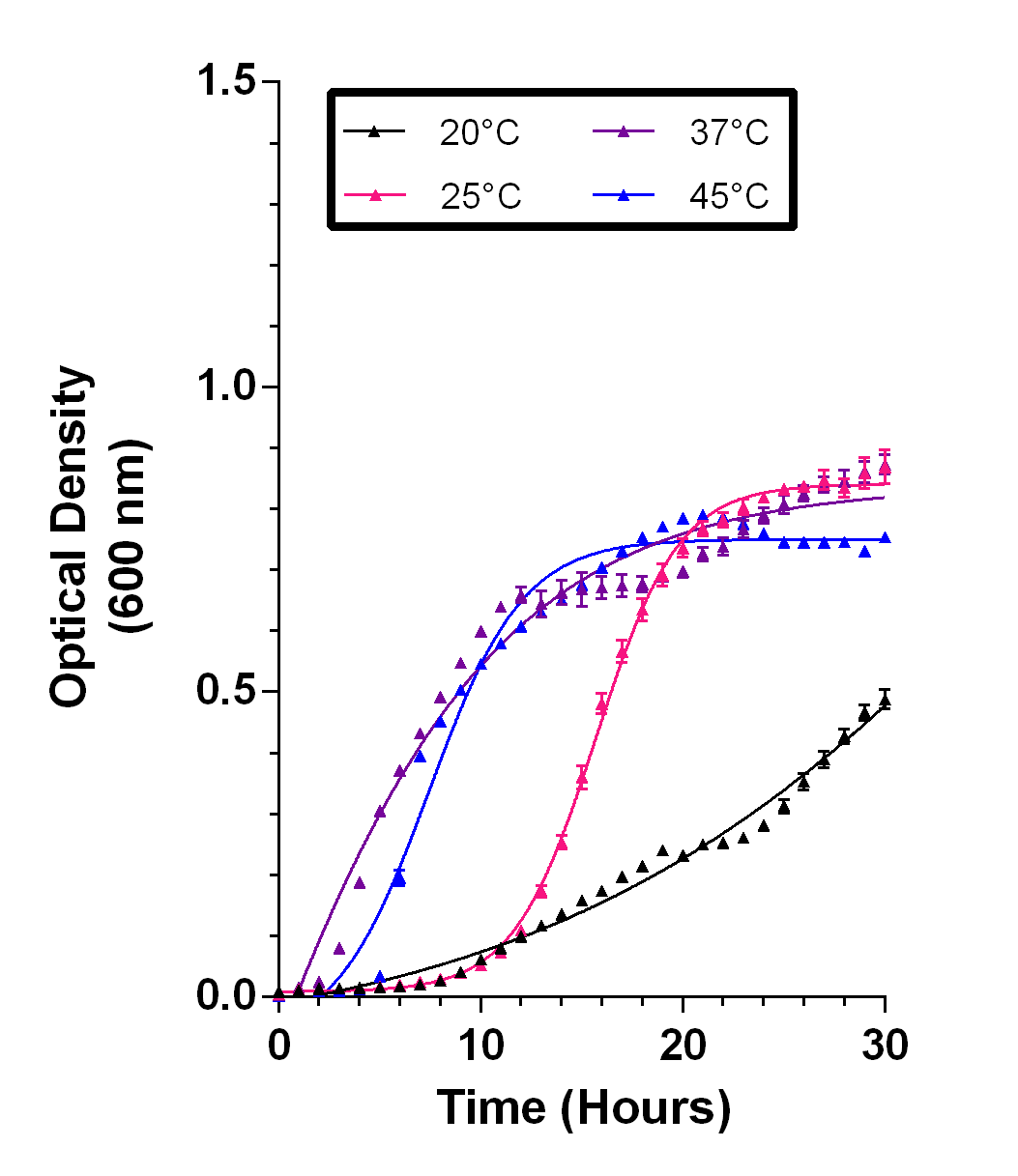


B)


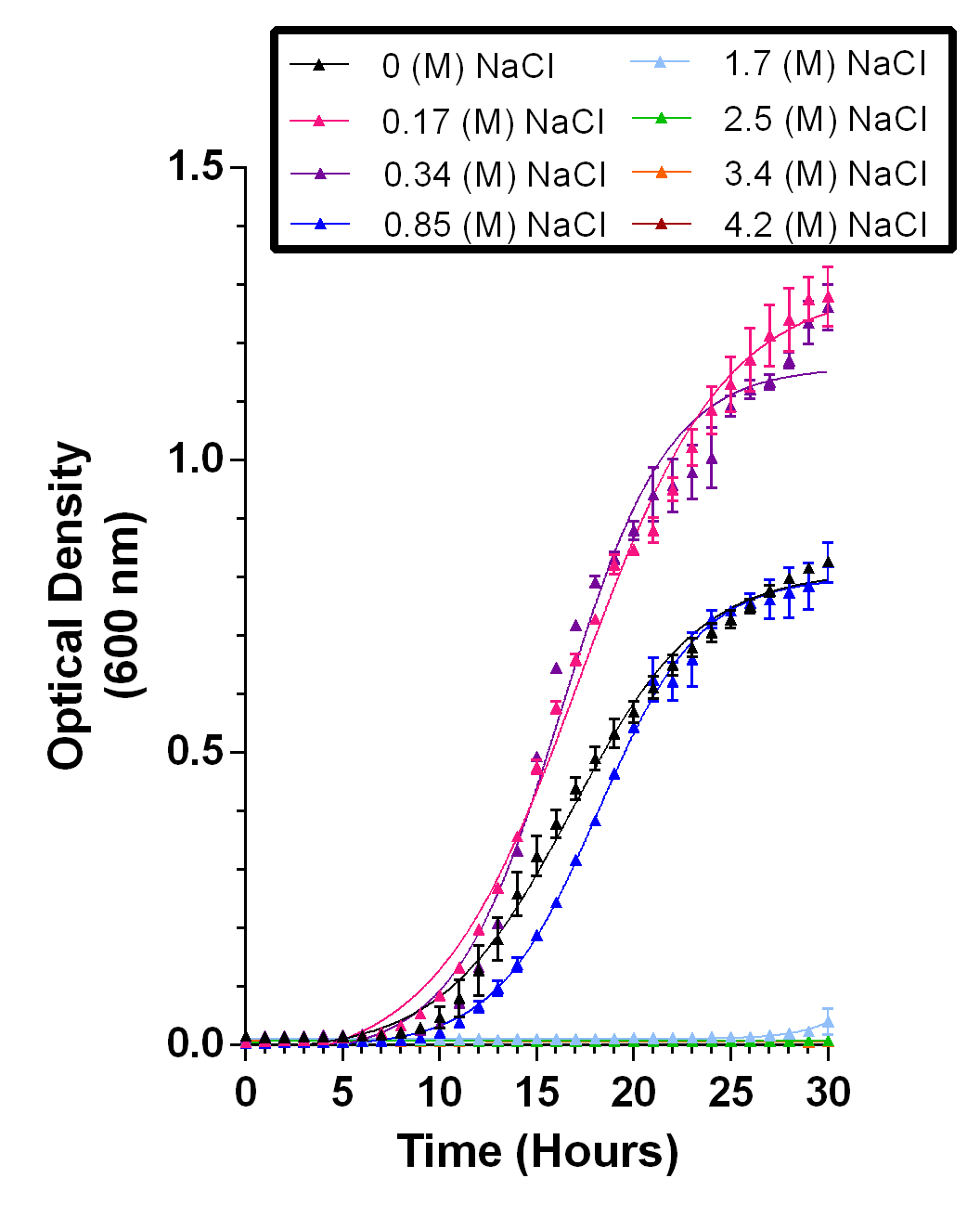


C)


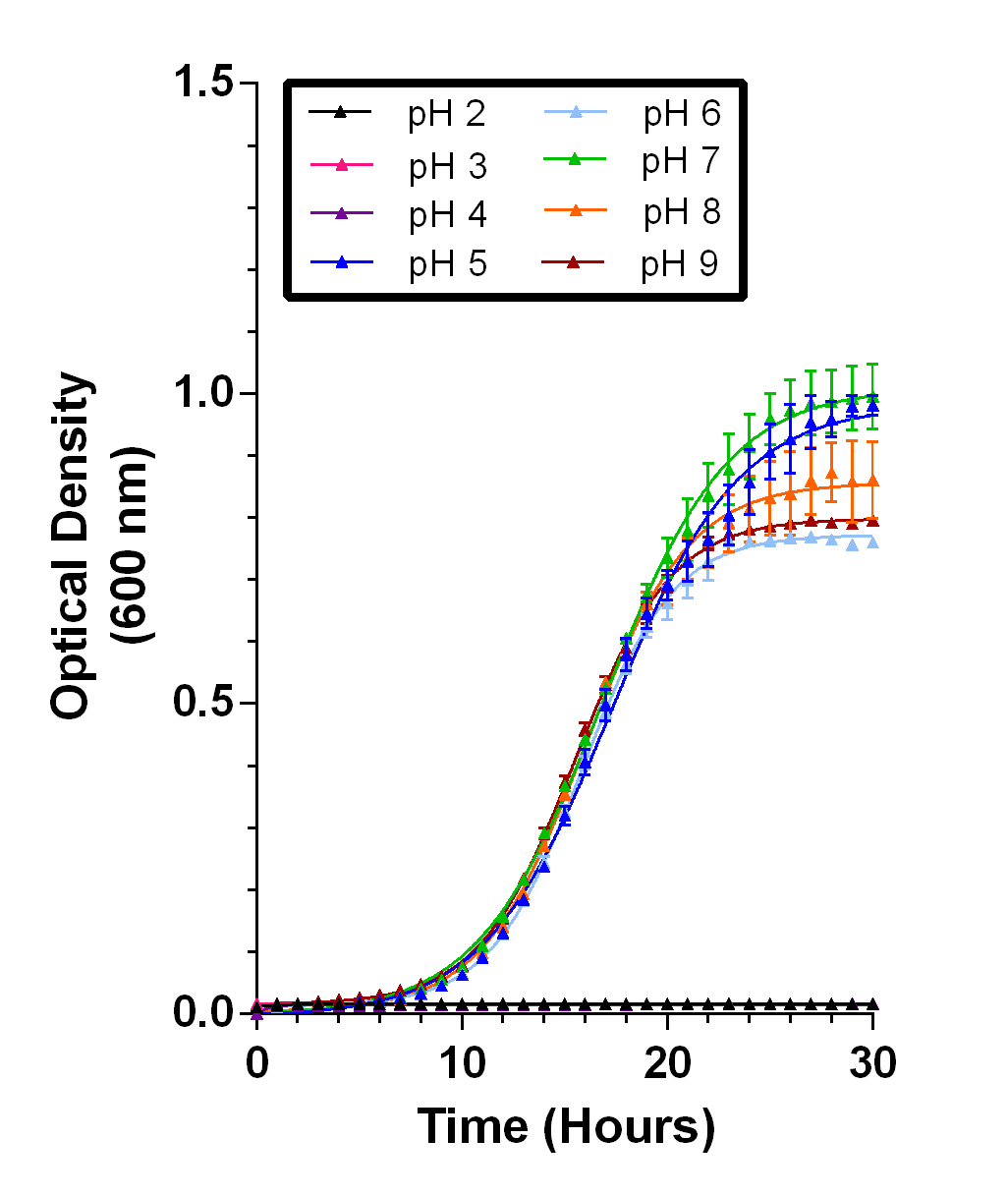


**Supplementary Figure S2**. Microbial growth of LIBR003 strain under different experimental condition: A) Temperature, B) NaCl- growth and C) pH. Values are mean ± standard error of the mean of experiments (SEM). The trend lines are represented as solid lines.

## Supplementary Tables

**Table S1.** Generation time of isolates according at temperature. Values are mean ± standard error of the mean of experiments (SEM).

| Temperature (°C) | Strain | |
| --- | --- | --- |
|  | LIBR002 | LIBR003 |
|  | Generation Time (Hours) | |
| 20 | 4.64 ± 0.41 | 5.08 ± 0.13 |
|  |  |  |
| 25 | 4.63 ± 0.28 | 3.91 ± 0.32 |
|  |  |  |
| 37 | 3.12 ± 0.06 | 3.52 ± 0.24 |
|  |  |  |
| 45 | 4.14 ± 0.18 | 3.76 ± 0.18 |

**Table S2.** Generation time of isolates according at NaCl- growth. Values are mean ± standard error of the mean of experiments (SEM).

| NaCl concentrations  (M) | Strain | |
| --- | --- | --- |
|  | LIBR002 | LIBR003 |
|  | Generation Time (Hours) | |
| 0 | 4.65 ± 0.18 | 5.34 ± 0.21 |
|  |  |  |
| 0.17 | 4.07 ± 0.05 | 3.77 ± 0.20 |
|  |  |  |
| 0.34 | 3.89 ± 0.32 | 4.59 ± 0.20 |
|  |  |  |
| 0.85 | 3.97 ± 0.20 | 3.89 ± 0.06 |
|  |  |  |
| 1.7 | 6.01 ± 0.19 | 16.38 ± 3.96 |
|  |  |  |
| 2.5 | ND | ND |
|  |  |  |
| 3.4 | ND | ND |
|  |  |  |
| 4.2 | ND | ND |

ND not detected.

**Table S3.** Generation time of isolates according at pH. Values are mean ± standard error of the mean of experiments (SEM).

| pH | Strain | |
| --- | --- | --- |
|  | LIBR002 | LIBR003 |
|  | Generation Time (Hours) | |
| 2 | ND | ND |
|  |  |  |
| 3 | 6.45 ± 1.11 | ND |
|  |  |  |
| 4 | 8.92 ± 1.37 | ND |
|  |  |  |
| 5 | 4.94 ± 0.13 | 4.87 ± 0.07 |
|  |  |  |
| 6 | 4.46 ± 0.08 | 4.96 ± 0.07 |
|  |  |  |
| 7 | 5.01 ± 0.07 | 5.01 ± 0.16 |
|  |  |  |
| 8 | 4.84 ± 0.14 | 5.02 ± 0.08 |
|  |  |  |
| 9 | 4.95 ± 0.15 | 5.19 ± 0.23 |

ND not detected.

**Table S4.** Metabolic profiles of isolates from lithium brines. (+ Positive; ± Intermediate; - Negative).

| **Carbon sources** | Strain | |
| --- | --- | --- |
|  | LIBR002 | LIBR003 |
|  | Reaction | |
| 3-Methyl Glucose | **-** | **-** |
| Acetic Acid | **-** | **-** |
| Acetoacetic Acid | **-** | **-** |
| Bromo Succinic Acid | **-** | ± |
| Citric Acid | **+** | **+** |
| D-Arabitol | **-** | **-** |
| D-Aspartic Acid | **-** | **+** |
| D-Cellobiose | ± | ± |
| D-Fructose | ± | **-** |
| D-Fructose-6-PO_4_ | **-** | **-** |
| D-Fucose | **-** | **-** |
| D-Galactose | **-** | **-** |
| D-Galacturonic Acid | ± | **-** |
| D-Gluconic Acid | ± | **-** |
| D-Glucose-6-PO_4_ | **-** | **-** |
| D-Lactic Acid Methyl Ester | **-** | **-** |
| D-Malic Acid | **-** | **-** |
| D-Maltose | **-** | **-** |
| D-Mannitol | ± | ± |
| D-Mannose | ± | **-** |
| D-Melibiose | **-** | **-** |
| D-Saccharic Acid | **-** | **-** |
| D-Salicin | ± | ± |
| D-Serine | **-** | **-** |
| D-Sorbitol | ± | **-** |
| D-Trehalose | ± | ± |
| D-Turanose | ± | **-** |
| Dextrin | **-** | **-** |
| Formic Acid | **-** | **-** |
| Gelatin | **-** | **-** |
| Gentibiose | ± | ± |
| Glucuronamide | **-** | **-** |
| Glycerol | ± | **-** |
| Glycyl-L-Proline | **-** | **-** |
| Inosine | **-** | **-** |
| L-Arginine | **-** | **-** |
| L-Aspartic Acid | ± | **+** |
| L-Butyric Acid | **-** | - |
| L-Fucose | **-** | **-** |
| L-Galactonic Acid Lactone | ± | **-** |
| L-Glutamic Acid | ± | ± |
| L-Histidine | **-** | **-** |
| L-Lactic Acid | **+** | **-** |
| L-Malic Acid | **+** | **+** |
| L-Pyroglutamic Acid | **-** | **-** |
| L-Rhamnose | **-** | **-** |
| L-Serine | **-** | ± |
| Methyl Piruvate | **-** | ± |
| Mucic Acid | **-** | **-** |
| myo-Inositol | **-** | **-** |
| N-Acetyl-D-Glucosamine | **-** | **-** |
| N-Acetyl-Neuraminic Acid | **-** | **-** |
| N-Acetyl-β-D-Mannosamine | **-** | **-** |
| N-D-Galactosamine | **-** | **-** |
| Pectin | ± | ± |
| Propionic Acid | **-** | **-** |
| Quinic Acid | **-** | **+** |
| Raffinose | **-** | **-** |
| Stachyose | **-** | **-** |
| Sucrose | ± | **-** |
| Tween 40 | **-** | **-** |
| α- D-Glucose | ± | ± |
| α- Hydroxy-Butyric Acid | **-** | **-** |
| α- Keto-Glutaric Acid | **-** | **-** |
| α-D-Lactose | **-** | **-** |
| α-Keto-Butyric Acid | **-** | **-** |
| β- Hydroxy-D | **-** | **-** |
| β- Methyl-D-Glucoside | ± | ± |
| γ-Amino-Butryric Acid | **-** | ± |
| ρ – Hydroxy-Phenylacetic Acid | **-** | **-** |
| **Chemical sensitivity** |  |  |
| Aztreonam | **+** | **+** |
| Fusidic Acid | **-** | **-** |
| Guanidine HCl | **+** | **+** |
| Lincomycin | **-** | ± |
| Lithium Chloride | **+** | **+** |
| Minocycline | **-** | **-** |
| NaCl 1% | **+** | **+** |
| NaCl 4% | **+** | **+** |
| NaCl 8% | **+** | **+** |
| Nalidixic Acid | **-** | **-** |
| Niaproof 4 | **-** | **-** |
| pH 5 | **+** | **-** |
| pH 6 | **+** | **+** |
| Potassium Tellurite | **+** | **+** |
| Rifamycin SV | **-** | **+** |
| Sodium Bromate | **+** | ± |
| Sodium Butyrate | **+** | **+** |
| Sodium Lactate 1% | **+** | **+** |
| Tetrazolium Blue | **-** | **-** |
| Tetrazolium Violet | **-** | **-** |
| Troleandomycin | **-** | **-** |
| Vancomycin | **-** | **-** |

**Table S5.** Enzymatic Activity Profile of isolates from lithium brines. (+ Positive; - Negative).

| **Reactions-Enzymes** | Strain | |
| --- | --- | --- |
|  | LIBR002 | LIBR003 |
|  | Reaction | |
| Alkaline phosphatase | - | - |
| Esterase | + | + |
| Esterase Lipase | + | + |
| Lipase | - | - |
| Leucine arylamidase | + | - |
| Valine arylamidase | + | + |
| Cystine arylamidase | + | - |
| Trypsin | - | + |
| α-chymotrypsin | - | + |
| Acid phosphatase | + | - |
| Naphthol-AS-BI-phosphohydrolase | + | + |
| α-galactosidase | - | - |
| β-galactosidase | - | - |
| β-glucuronidase | - | - |
| α-glucosidase | - | - |
| β-glucosidase | + | - |
| N-acetyl-β-glucosaminidase | + | - |
| α-mannosidase | - | - |
| α-fucosidase | - | - |
